# Exploratory multi-omics analysis of gut microbiota and fecal metabolites in relation to serum S-equol levels in older adults with osteoporosis from a tropical community: a pilot study

**DOI:** 10.3389/fnut.2026.1784894

**Published:** 2026-02-18

**Authors:** Wenting Cao, Rui Li, Hanxiang Zhang, Tianxia Zhang, Hongxin Pan, Wen Sun, Lingqi Wang, Jiashu Ke, Jindong Ding Petersen, Ping Zhang

**Affiliations:** 1 Key Laboratory of Tropical Translational Medicine of Ministry of Education, School of Public Health, Hainan Academy of Medical Sciences, Hainan Medical University, Haikou, Hainan, China; 2 Department of Nuclear Medicine, First Affiliated Hospital of Hainan Medical University, Haikou, Hainan, China; 3 Department of Laboratory Diagnosis, Fifth Affiliated Hospital of Harbin Medical University, Daqing, Heilongjiang, China; 4 Department of Public Health, University of Copenhagen, Copenhagen, Denmark; 5 Department of Public Health, University of Southern Denmark, Odense, Denmark

**Keywords:** bone health, gut microbiota, metabolomics, osteoporosis, S-equol

## Abstract

**Background:**

Osteoporosis (OP) is a multifactorial skeletal disorder influenced by host metabolism, inflammation, and gut microbiota-derived metabolites such as S-equol. However, the interplay between intestinal microbiota, S-equol production, and host metabolic profiles in OP remains incompletely understood.

**Objective:**

To conduct a preliminary multi-omics investigation integrating metagenomic and metabolomic analyses to identify gut microbiota and metabolite biomarkers associated with serum S-equol levels in older adults with OP.

**Methods:**

A cross-sectional study was conducted in 39 community-dwelling adults aged ≥50 years in Haikou, China. Participants were grouped into OP and control groups based on lumbar spine T-scores, using a cut-off value of ≤ − 2.5 to define osteoporosis. Serum biomarkers (S-equol, inflammatory cytokines, oxidative stress indicators) were assessed by ELISA. Fecal samples underwent metagenomic sequencing and untargeted metabolomics. LEfSe, Spearman correlation, machine learning, and KEGG enrichment were used to explore microbiota-metabolite-bone health axes.

**Results:**

Serum S-equol levels were significantly lower in the OP group compared to controls (3,561 ± 304 vs. 3,855 ± 469 pg/mL, *p* = 0.026), whereas most inflammatory markers were comparable between groups, apart from a modest increase in IL-1*β* in OP. Metagenomic analysis revealed a lower relative abundances of key SCFA-producing taxa in OP (e.g., *Faecalibacterium prausnitzii*, *Roseburia hominis*, *Bacteroides uniformis*). Metabolomic profiling identified distinct alterations in amino acid and tryptophan pathways, with KEGG analysis highlighting disruptions in glycerophospholipid, glycine-serine–threonine, and choline metabolism. Discriminative metabolites (e.g., Gln-Val-Ile-Asp., 5-oxooctanoic acid) showed diagnostic potential (AUC > 0.75). S-equol levels positively correlated with these beneficial microbes and with amino acid-related metabolites (e.g., D-tryptophan, 3-indoleacrylic acid, N-methylglutamate). Network and heatmap analyses illustrated differences in microbial-metabolite association patterns between groups.

**Conclusion:**

In conclusion, low levels of serum S-equol in older adults with osteoporosis were associated with distinct changes in gut microbiota composition and fecal metabolic profiles in this pilot study.

## Highlights

- Serum S-equol levels were significantly lower in older adults with osteoporosis from a tropical community.
- Osteoporosis was associated with differences in gut microbiota and fecal metabolic profiles.
- Lower serum S-equol levels in osteoporosis were associated with lower relative abundances of SCFA-producing bacteria and tryptophan-related metabolites.

## Introduction

Osteoporosis (OP) is a major public health concern worldwide, particularly in aging populations, characterized by progressive bone loss and increased susceptibility to fragility fractures (1, 2). In China, the 2018 national report identified OP as a major health issue among individuals aged 50 and above, particularly affecting older women. Nationally, OP affects approximately 19.2% of this population, with prevalence reaching 29.13% in females and 6.46% in males (3, 4). With the rapid growth of the elderly population, the prevalence of OP continues to rise, imposing substantial medical, economic, and social burdens (5). In older adults, osteoporotic fractures are closely associated with disability, loss of independence, and reduced quality of life, underscoring the urgent need for effective prevention strategies. Although pharmacological treatments are available, their long-term use may be limited by adverse effects, cost, and restricted accessibility, especially in community-dwelling older adult populations (6, 7). Consequently, nutritional approaches have gained increasing attention as practical and sustainable approaches for OP prevention and early intervention. Adequate intake of bone-related nutrients and bioactive dietary components plays a crucial role in maintaining skeletal health throughout aging. Among these, soy isoflavones have emerged as a prominent class of plant-derived phytoestrogens with potential bone-protective effects. Structurally similar to endogenous estrogens, soy isoflavones can interact with estrogen receptors and have been suggested to modulate bone turnover, inflammation, and oxidative stress (8). Many epidemiological and experimental evidences support the beneficial role of soy-rich diets in preserving bone mass, particularly in populations with habitual soy consumption, highlighting the importance of dietary factors and functional nutrients in mitigating age-related bone loss (9, 10).

S-equol, a gut microbiota-derived metabolite of soybean isoflavones, particularly daidzein, exhibits stronger estrogenic activity, higher bioavailability, and greater antioxidant capacity than its precursor compounds. By preferentially binding to estrogen receptor-*β*, S-equol can regulate osteoblast and osteoclast activity and thereby influence bone remodeling (11). Accumulating evidence from both human and animal studies suggests that higher endogenous S-equol levels are associated with increased bone mineral density (BMD) and a reduced risk of bone loss or osteoporotic fractures, especially in populations with habitual soy consumption. Experimental studies further indicate that S-equol can attenuate estrogen deficiency-induced bone loss, supporting its protective role in skeletal health (12–14). Notably, only a subset of individuals are capable of producing S-equol, a phenomenon that is largely determined by gut microbiota composition rather than dietary isoflavone intake alone (15). Several key equol-producing bacteria have been identified, which possess the enzymatic capacity to convert daidzein into S-equol, and the abundance and functional activity of these bacteria are considered critical determinants of an individual’s equol producer status (16). These findings highlight the importance of microbial metabolic capacity in mediating the biological effects of dietary phytoestrogens.

Because S-equol production depends on specific microbial metabolic functions and is measurable in circulation, joint analysis of serum S-equol, gut microbiota composition, and fecal metabolomic profiles enables a more integrated assessment of microbial metabolism and host exposure to bioactive compounds, with relevance to bone health. Against this background, the gut microbiota, beyond its role in S-equol production, has been increasingly recognized as a central regulator of bone physiology, giving rise to the concept of the gut-bone axis (17, 18). Gut microorganisms influence bone health through the production of a variety of metabolites, including short-chain fatty acids (SCFAs), amino acids, and tryptophan-derived metabolites, which are involved in the regulation of inflammation, oxidative stress, immune responses, and osteoblast–osteoclast coupling (19, 20). Dysbiosis of the gut microbiota and alterations in these metabolic pathways have been increasingly linked to imbalanced bone remodeling during aging, showing that the gut ecosystem represents a promising target for OP prevention and intervention (21, 22). In this context, integrating metabolomic profiling with gut microbiota analysis offers a more comprehensive understanding of how microbial-derived metabolites contribute to host bone metabolism, extending beyond taxonomic characterization alone.

Despite the burgeoning interest in the gut-bone axis, several important knowledge gaps remain. To date, few studies have systematically examined the relationships among S-equol levels, gut microbiota composition, and fecal metabolomic profiles in osteoporotic populations. Consequently, evidence integrating diet-derived metabolites with microbial metabolism in the context of bone health remains limited. Moreover, most published studies in this field have focused primarily on postmenopausal women (23, 24), leaving a knowledge gap regarding the broader older adult population, including older men. In addition, limited attention has been given to whether tropical dietary patterns and environmental contexts influence equol-producing capacity, gut microbial structure, and metabolic features related to bone health. To address these gaps, the present study was conducted in a community-dwelling older adult population in Hainan, a tropical region in China, by using an integrated multi-omics approach. We aimed to investigate the associations between serum S-equol levels, gut microbiota composition, and fecal metabolic profiles, and to identify potential microbiota- and nutrition-related metabolites associated with OP. This work provides novel insights into geographically relevant microbial-metabolic characteristics and may contribute to the development of personalized strategies for OP prevention and management.

## Materials and methods

### Study design and participants

This research employed a cross-sectional research design. From August to October 2024, community-dwelling adults were recruited from Xinglinyuan Community in Haikou City, Hainan Province, China, through a combination of convenience sampling and volunteer recruitment.

Inclusion criteria were as follows: (1) aged ≥ 50 years; (2) residence in the target community for more than 6 months; (3) voluntary participation with a clear understanding of the study objectives; (4) ability to communicate effectively and independently complete all required assessments. Exclusion criteria included: (1) a history of lumbar spine surgery that could interfere with accurate BMD measurement; (2) use of antibiotics within the previous 3 months; (3) severe physical disabilities or terminal illnesses that limited participation; (4) serious mental disorders preventing completion of the questionnaire; (5) refusal to provide informed consent or withdrawal during data collection. The study protocol was approved by the Ethics Committee of Hainan Medical University (Approval No. HYLL-2023-098). All participants provided written informed consent prior to enrollment, and the study was conducted in accordance with the principles of the Declaration of Helsinki. The study finally obtained 39 older adult participants with 19 and 20 in the OP group and control group, respectively.

### Blood sample collection and biochemical analysis

Fasting venous blood samples were collected from all participants in the morning under standardized conditions. Approximately 5 mL of whole blood was drawn into vacuum tubes without anticoagulants and centrifuged at 3000 rpm for 10 min to separate the serum.

Serum levels of S-equol were measured using a commercial ELISA kit (Shanghai Enzyme-Linked Biotechnology Co., Ltd., China). Inflammatory cytokines, including interleukin-6 (IL-6), IL-1*β*, tumor necrosis factor-*α* (TNF-*α*), monocyte chemoattractant protein-1 (MCP-1), and high-sensitivity C-reactive protein (hs-CRP), as well as malondialdehyde (MDA) and total antioxidant status (TAS), were quantified using ELISA kits purchased from Nanjing Boyan Biotechnology Co., Ltd., China, in accordance with the manufacturer’s protocols. All assays were performed in duplicate, and the mean values were used for subsequent statistical analyses. According to the manufacturer’s instructions, the intra-assay and inter-assay coefficients of variation (CVs) for the serum S-equol assay were <8 and <10%, respectively. The oxidative stress index (OSI) was calculated as the ratio of MDA to TAS (25).

### Bone mineral density (BMD) measurement and participant classification

BMD of the lumbar spine (L1–L4 vertebrae) was measured for all participants using dual-energy X-ray absorptiometry (DXA) scanner (GE Lunar Prodigy, USA). Prior to scanning, participants were instructed to remove any metal-containing items that might interfere with image quality, and they were positioned in a standard supine posture on the DXA examination table to align the lumbar spine with the scanner’s detection range. Based on the World Health Organization (WHO) diagnostic criteria, participants were classified into two groups according to their T-scores: the OP group with T-scores ≤ − 2.5 (26), and the control group with T-scores > − 2.5. The measurements were performed at the Department of Clinical Laboratory, the First Affiliated Hospital of Hainan Medical University, and all scans were performed on the same device to minimize inter-instrument variability and ensure data consistency.

### Fecal sample collection and DNA extraction

Fresh fecal samples were collected from each participant using sterile collection tubes and immediately transported to the laboratory in a dry ice box, where they were stored at – 80 °C until further processing. Total microbial DNA was extracted from frozen fecal samples using the QIAamp PowerFecal Pro DNA Kit (QIAGEN, China), following the manufacturer’s instructions. DNA concentration and purity were assessed using a NanoDrop One spectrophotometer (Thermo Fisher Scientific, USA).

### Metagenomic sequencing and analysis

Total DNA extracted from fecal samples was used for whole-metagenome shotgun sequencing. Sequencing libraries were prepared following standard protocols and sequenced on the Illumina NovaSeq XPlus platform (Illumina Inc., USA), generating 150 bp paired-end reads. Raw sequencing data were subjected to quality control using FastQC and Trimmomatic, with removal of low-quality reads and host DNA contamination. Clean reads were then assembled using MEGAHIT and annotated using the MetaPhlAn3 pipeline for taxonomic profiling at the species level. Functional annotation was performed based on the Kyoto Encyclopedia of Genes and Genomes (KEGG) and EggNOG databases using HUMAnN3. Relative abundances of microbial taxa and functional pathways were calculated and compared between the control and OP groups. Alpha-diversity and beta-diversity metrics were computed using QIIME2. Differentially abundant species and functions were identified using LEfSe and STAMP, with a Linear Discriminant Analysis (LDA) score threshold set at 2.0 and *p* < 0.05 considered statistically significant.

### Untargeted metabolomics analysis

Untargeted metabolomic profiling of fecal samples was conducted by LC–MS/MS using an Ultra-High-Performance Liquid Chromatography (UHPLC) system (Thermo Dionex Ultimate 3,000) coupled with a Q Exactive HF-X Orbitrap Mass Spectrometer (Thermo Fisher Scientific, USA). Metabolite extraction was performed using a methanol-based protocol, followed by centrifugation and supernatant collection. Samples were analyzed in both positive and negative electrospray ionization (ESI) modes.

Raw MS data were processed using Compound Discoverer (v3.1, Thermo, USA) for peak detection, alignment, and normalization. Metabolite identification was based on accurate mass-to-charge ratio (m/z), retention time, and MS/MS fragmentation patterns by searching against HMDB, KEGG, and mzCloud databases. Multivariate statistical analyses including principal component analysis (PCA) and orthogonal partial least squares discriminant analysis (OPLS-DA) were performed using SIMCA (v14.1) to visualize group separation and detect differential metabolites.

Differential metabolites between OP and control groups were identified based on VIP > 1, fold change ≥ 1.2, and adjusted *p* < 0.05. KEGG pathway enrichment analysis of differential metabolites was conducted to explore their potential biological relevance, particularly focusing on metabolic pathways associated with bone metabolism, inflammation, and microbial-host interactions.

### Multi-omics correlation analysis

To explore the relationship between the gut microbiota and fecal metabolites, multi-omics correlation analysis was conducted using the Spearman correlation method. First, the relative abundance of differential gut microbial species obtained from metagenomic sequencing and the normalized peak intensities of differential fecal metabolites identified through untargeted metabolomics were selected. Spearman’s rank correlation coefficients were calculated between microbial species and metabolites across all participants.

Significant correlations were defined based on both correlation coefficient thresholds (|rho| > 0.5) and statistical significance (*p* < 0.05). The resulting correlation matrix was visualized as a heatmap using the “pheatmap” package in R (version 4.2). Furthermore, microbial-metabolite interaction networks were constructed and visualized using Cytoscape software (version 3.9.1) to better elucidate the potential biological interactions between dominant gut taxa and their associated metabolites.

### Statistical analysis

Descriptive statistics were applied to summarize demographic variables and clinical characteristics. Continuous variables were tested for normality using the Shapiro–Wilk test. Normally distributed data were presented as means ± standard deviations (SD), whereas non-normally distributed data were presented as medians with interquartile ranges (IQR). Between-group comparisons were performed using independent sample t-tests or Mann–Whitney U tests, as appropriate. Categorical variables were reported as counts and percentages and compared using Fisher’s exact tests. All statistical analyses were performed using SPSS version 20.0 (IBM Corp., Armonk, NY, USA), and R software version 4.2. *p* < 0.05 was considered statistically significant unless otherwise specified.

## Results

### Baseline characteristics and serum indicators between older adults with and without OP

The demographic characteristics and baseline clinical parameters of the OP and control groups are summarized in Table 1. No significant differences were observed between the two groups in terms of age, sex, smoking status, alcohol consumption, or other sociodemographic variables (all *p* > 0.05), showing that the groups were well matched and comparable.

**Table 1:** The demographic characteristics and serum indicators of participants in the OP and control groups.

Variables	OP (*n* = 19)	Control (*n* = 20)	*p*
Age, years	64.1 ± 6.3	64.5 ± 6.6	0.848
Sex, *n* (%)			0.224
Female	14 (73.7)	11 (55.0)	
Male	5 (26.3)	9 (45.0)	
Marriage, *n* (%)			0.184
Married	14 (73.7)	18 (90.0)	
Other	5 (26.3)	2 (10.0)	
Education years, *n* (%)			0.755
≤6	5 (26.3)	3 (15.0)	
7–9	4 (21.1)	5 (25.0)	
>9	10 (52.6)	12 (60.0)	
Income/moth, Yuan, *n* (%)			0.316
≤2000	9 (47.4)	5 (25.0)	
2001–5,000	5 (26.3)	9 (45.0)	
>5,000	5 (26.3)	6 (30.0)	
Smoking, *n* (%)	5 (26.3)	1 (5.0)	0.091
Alcohol, *n* (%)	5 (26.3)	2 (10.0)	0.235
Body mass index, kg/m^2^	23.1 ± 2.2	25.5 ± 3.2	**0.009**
Serum indicators			
S-equol, pg/mL	3,561 ± 304	3,855 ± 469	**0.026**
IL-6, pg/mL	4.27 (1.08–5.12)	4.46 (4.12–4.57)	0.531
IL-1*β*, pg/mL	45.0 ± 16.5	35.8 ± 10.3	**0.043**
TNF-*α*, pg/mL	21.6 (17.3–35.5)	20.5 (15.2–30.6)	0.588
MCP-1, pg/mL	379 (335–415)	379 (349–435)	0.627
hs-CRP, mg/L	6.21 (5.56–7.07)	6.30 (4.89–7.94)	0.792
MDA, nmol/mL	2.26 (1.94–2.26)	2.58 (2.26–3.15)	0.127
TAS, μmol Trolox/mL	0.80 (0.62–1.09)	0.75 (0.66–1.11)	0.945
OSI, ×10^−3^	3.15 (1.89–3.83)	3.64 (2.31–4.59)	0.283
Lumbar spine BMD, g/cm^2^			
L1	0.69 ± 0.06	0.84 ± 0.13	**<0.001**
L2	0.71 ± 0.07	0.88 ± 0.12	**<0.001**
L3	0.79 ± 0.07	0.95 ± 0.12	**<0.001**
L4	0.78 ± 0.07	0.96 ± 0.08	**<0.001**
L1-L4	0.75 ± 0.03	0.92 ± 0.08	**<0.001**
T-scores	−2.88 ± 0.31	−1.35 ± 0.08	**<0.001**

Data are presented as mean ± standard deviation, median (interquartile range), or number (%) as appropriate. *p* values were calculated using independent sample *t* test, Mann–Whitney *U* test, or Fisher’s exact test, depending on data type and distribution. *p* values in bold indicate statistical significance (*p* < 0.05).

OP, Osteoporotic group; BMD, bone mineral density; IL, interleukin; TNF-*α*, tumor necrosis factor *α*; MCP-1, monocyte chemoattractant protein-1; hs-CRP, high-sensitivity C-reactive protein; MDA, malondialdehyde; TAS, total antioxidant status; OSI, oxidative stress index.

OSI was calculated as the ratio of MDA to TAS.

L1–L4 refers to the mean BMD of the first to fourth lumbar vertebrae measured by dual-energy X-ray absorptiometry (DXA).

Of particular interest, S-equol was significantly lower levels in the OP group compared to controls (3,561 ± 304 vs. 3,855 ± 469 pg/mL, *p* = 0.026). In addition, IL-1*β* levels were significantly higher in the OP group than in the control group (45.0 ± 16.5 vs. 35.8 ± 10.3, *p* = 0.043), and indicated higher inflammation in individuals with OP.

Additionally, participants with OP showed significantly lower lumbar spine BMD values across all vertebrae (L1–L4) (all *p* < 0.001). While the average T-score in the OP group was markedly lower (−2.88 ± 0.31) than that in the control group (−1.35 ± 0.08, *p* < 0.001), consistent with the diagnostic classification of osteoporotic bone loss.

### Gut microbial diversity and genus-level composition in osteoporotic and non-osteoporotic older adults

**Figure 1:**
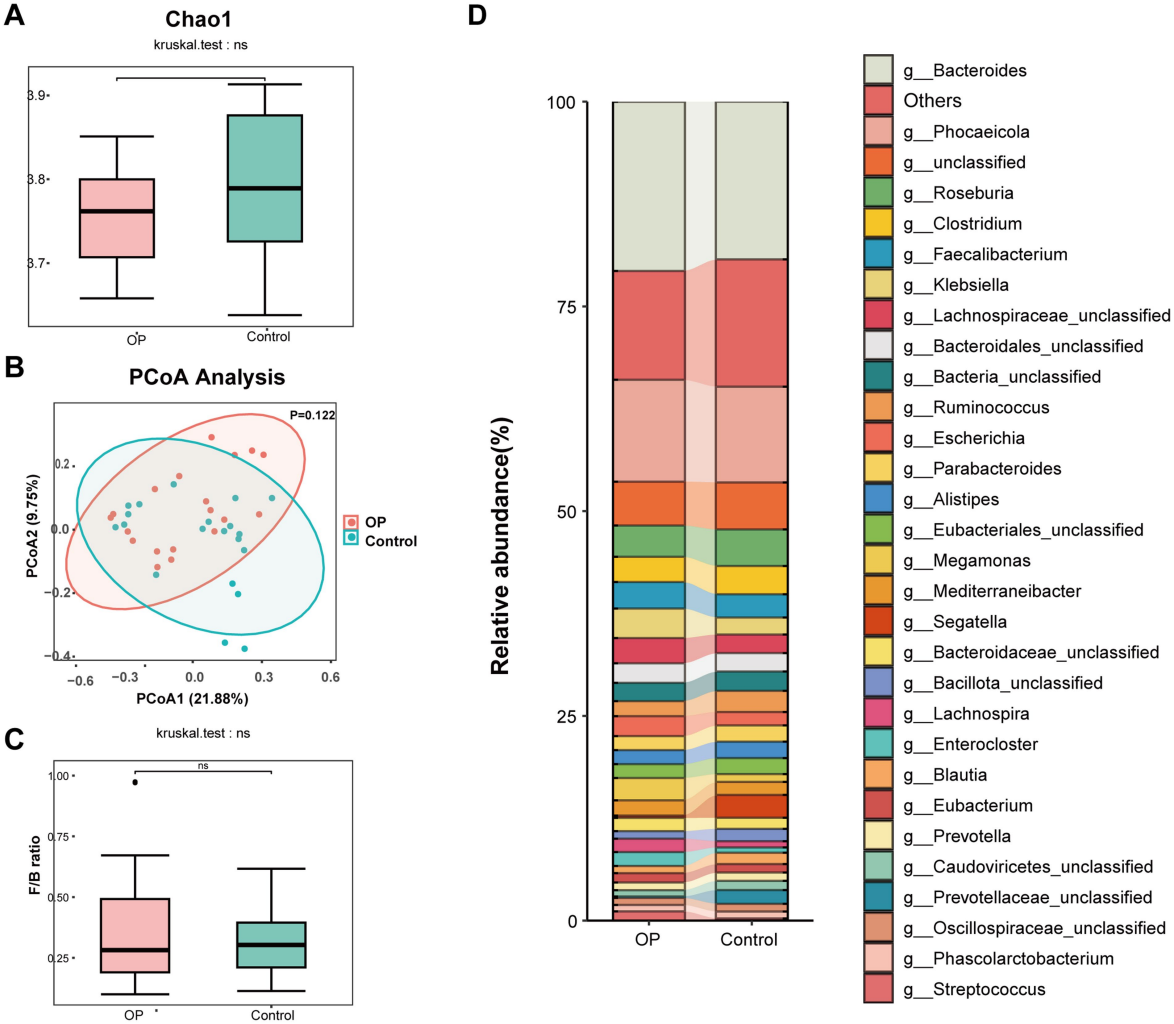
Gut microbial diversity and compositional differences between OP and control groups. **(A)** Box plot of alpha diversity measured by the Chao1 index, comparing microbial richness between the OP group and control group. **(B)** Principal coordinates analysis (PCoA) based on Bray–Curtis distance showing beta diversity between groups. **(C)** Comparison of the *Firmicutes/Bacteroidetes* (F/B) ratio between groups. **(D)** Stacked bar plot illustrating the relative abundance of dominant genera in the gut microbiota of each group.

To comprehensively characterize the gut microbial composition in all participants, whole-metagenome shotgun sequencing was performed on fecal samples. As shown in Figure 1A, there was no statistically significant differences in the Chao1 index between the OP and control groups, although the control group exhibited a slightly higher median value. In addition, Shannon and Simpson indices were also calculated to assess *α*-diversity, and no significant differences were observed between groups (Supplementary Figure S1). These findings suggest that the overall richness and evenness of the gut microbial communities were comparable between osteoporotic and non-osteoporotic individuals.

To assess inter-individual variability in gut microbial composition, beta diversity was analyzed using principal coordinates analysis (PCoA) based on Bray-Curtis distance metrics. The PCoA plot (Figure 1B) showed a partial separation trend between the OP and control groups along the primary coordinate axis (PCoA1, explaining 21.88% of the variance), although the difference was not statistically significant (*p* = 0.122). The 95% confidence ellipses illustrate considerable overlap between the two groups, showing that overall community structure was broadly similar, with some degree of compositional divergence. These findings imply that while *α*-diversity remained comparable, subtle differences in microbial community composition may exist between osteoporotic and non-osteoporotic individuals, potentially contributing to functional disparities in gut metabolism or host-microbiota interactions.

The Firmicutes-to-Bacteroidetes (F/B) ratio, a commonly used indicator of gut microbiota stability and metabolic state, has been associated with host energy metabolism, inflammation, and immune regulation. Therefore, we evaluated the F/B ratio as shown in Figure 1C, but no statistically significant difference was observed between the OP and control groups. To further characterize the gut microbial profiles of the study participants, genus-level taxonomic composition was assessed and compared between the OP and control groups. As shown in Figure 1D, both groups mainly comprised genera from the phyla *Bacteroidota* and *Bacillota*, with notable differences in the relative abundance of several key taxa. *Streptococcus* was relatively enriched in the OP group, while *Ruminococcus* exhibited slightly higher abundance in the control group. Other SCFA-producing genera such as *Faecalibacterium*, *Roseburia*, and *Blautia* were also more abundant in the control group.

### Discriminatory gut microbial features between older adults with and without OP

**Figure 2:**
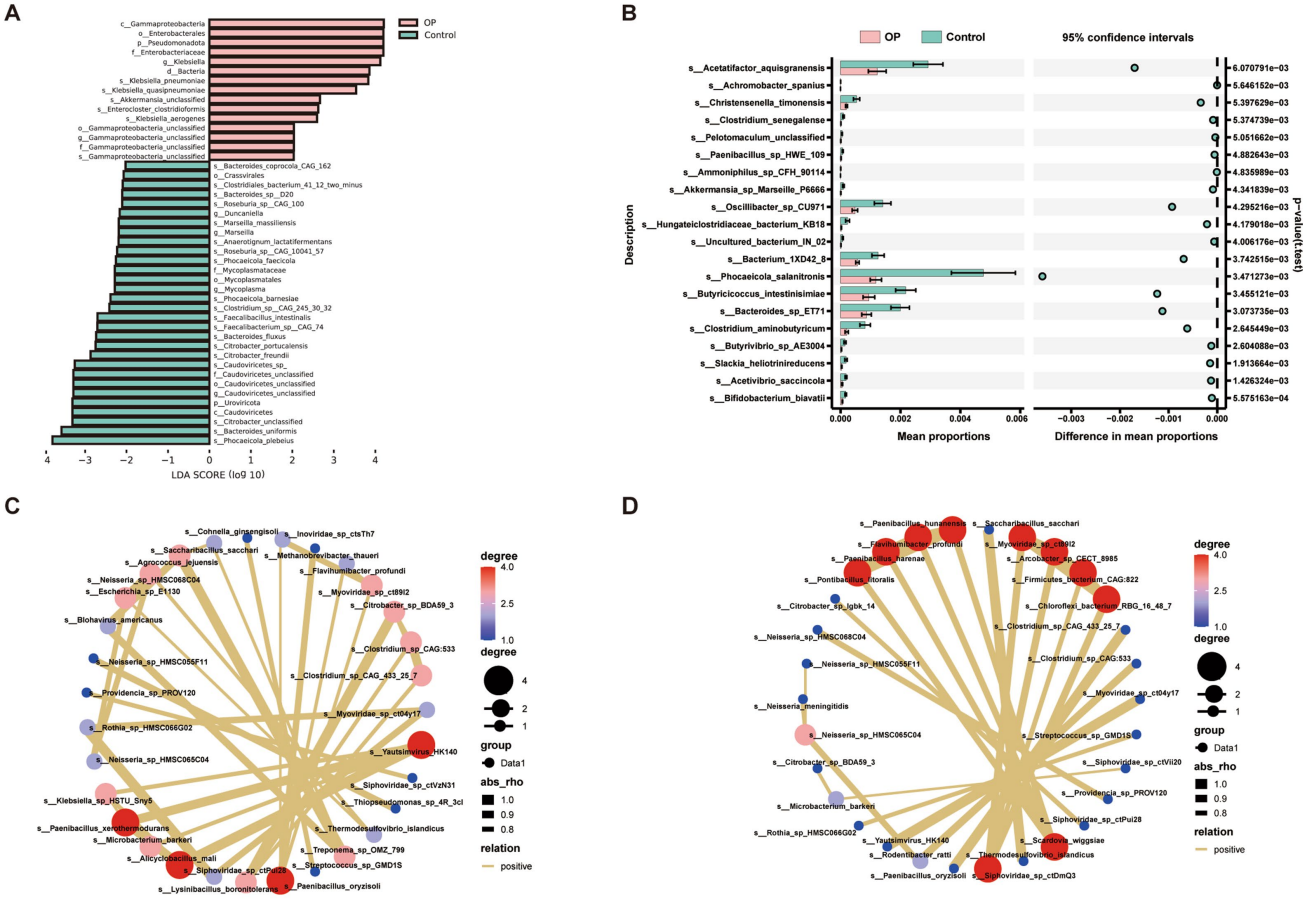
Differentially abundant microbial taxa and co-occurrence networks between OP and control groups. **(A)** Linear discriminant analysis effect size (LEfSe) identified bacterial taxa differentially abundant between the osteoporotic (OP, pink) and control (green) groups. Taxa with LDA scores (log_10_) > 2 and *p* < 0.05 were considered significant. **(B)** Extended error bar plot depicting the mean relative abundances and 95% confidence intervals for discriminative species-level taxa between groups. **(C,D)** Co-occurrence network analysis illustrating microbial interaction patterns in OP **(C)** and control **(D)** groups. Nodes represent microbial species, with size corresponding to degree (number of connections) and color indicating node centrality (red = higher degree). Edges denote significant positive correlations (Spearman’s *ρ* > 0.6, *p* < 0.05).

To identify key microbial taxa associated with OP, we employed both LEfSe analysis and species-level differential abundance testing. LEfSe analysis (Figure 2A) showed significant enrichment of taxa such as *Klebsiella pneumoniae*, *Gammaproteobacteria*, and *Akkermansia* in the OP group, while *Faecalibacterium prausnitzii*, *Roseburia*, and *Bacteroides uniformis* were more abundant in the control group. In parallel, we conducted unpaired t-tests at the species level to compare relative abundances between groups. As shown in Figure 2B, the top 20 species with the most significant differences displayed group-specific enrichment trends. Among these, species including *Phocaeicola salanitronis*, *Butyricicoccus intestinisimiae*, and *Bacteroides* sp. ET71 exhibited a higher abundance in the control group. Notably, *Akkermansia* sp. Marseille-P6666 and *Oscillibacter* sp. CU971, known to participate in lipid metabolism, anti-inflammatory functions and gut barrier integrity, were present at significantly lower relative abundances in individuals with OP compared with controls.

To further explore the ecological interactions among the differential species, we constructed group-specific microbial co-occurrence networks based on Spearman correlations (Figure 2C: Control; Figure 2D: OP). In these networks, red-colored nodes indicate species with higher degree centrality, such as *Clostridium* sp. CAG433 and *Paenibacillus oryzisoli*, showing that these taxa frequently co-occur with multiple other species and may serve as ecological hub species within the gut microbiome. Comparison of the two network structures revealed clear differences between the OP and control groups. Notably, species such as *Neisseria* sp. HMSC065C04 and *Providencia* sp. PROV120 appear in both networks; however, their connection strengths and interaction partners vary between the groups. Although these species are present across conditions, their roles within the microbial ecosystem may differ, possibly contributing distinctively to the development or modulation of OP.

### Functional characteristics of the gut microbiota and correlation analyses with host factors

**Figure 3:**
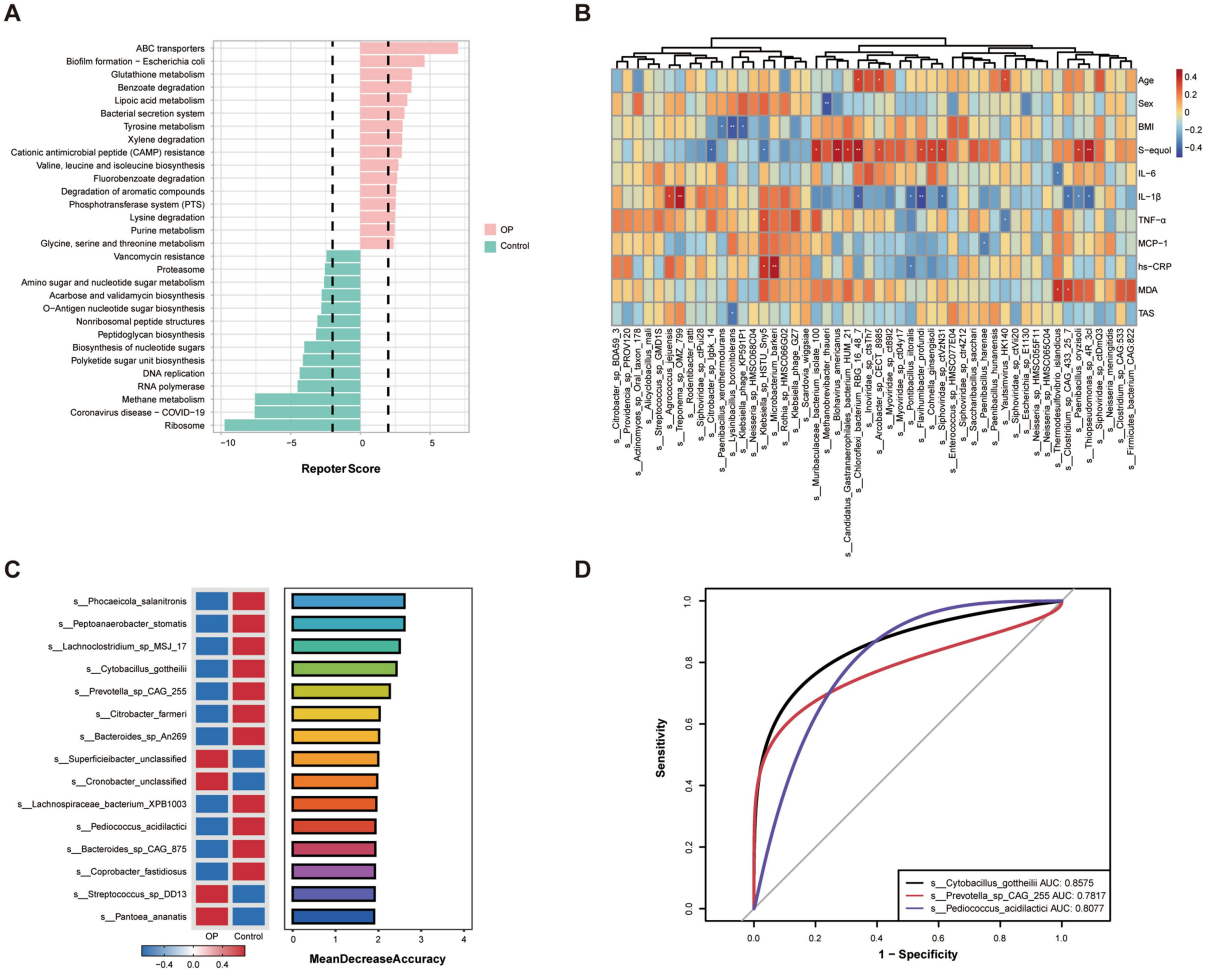
Functional characteristics of the gut microbiota and correlation analyses with host factors in OP and control groups. **(A)** Differential KEGG pathway prediction based on PICRUSt2 analysis. The reporter score plot illustrates the top significantly enriched pathways in the OP (pink) and control (green) groups. A reporter score threshold of ±1.65 (dashed lines) was used to identify significant functional differences. **(B)** Heatmap showing the Spearman correlation between significantly different microbial species and host factors, including age, sex, BMI, serum S-equol, inflammatory markers (IL-6, IL-1*β*, TNF-*α*, MCP-1, hs-CRP), and oxidative stress markers (MDA, TAS). Red and blue indicate positive and negative correlations, respectively. Asterisks denote statistically significant correlations (*p < 0.05, **p < 0.01). **(C)** Random forest model identifying the top 15 microbial species contributing to the discrimination between OP and control groups. The bar plot shows species importance ranked by mean decrease accuracy. The abundance distributions of key species across groups are shown on the left. **(D)** ROC curves evaluating the diagnostic performance of three representative microbial biomarkers.

To elucidate the functional implications of gut microbiota alterations in OP, we first performed microbial metabolic pathway analysis based on predicted KEGG orthologs (Figure 3A). The OP group exhibited significant upregulation in pathways related to ABC transporters, glutathione metabolism, and antimicrobial resistance, including bacterial secretion systems and biofilm formation. In contrast, the control group exhibited enrichment in core biosynthetic pathways such as ribosome biogenesis, RNA polymerase, and methanogenesis, showing distinct microbial functional associated with host bone health status.

Subsequently, a spearman correlation heatmap (Figure 3B) showed associations between host characteristics (age, sex, BMI), the phytoestrogen metabolite S-equol, serum biomarkers (e.g., IL-6, IL-1*β*, TNF-*α*, hs-CRP, MCP-1, MDA, TAS), and gut microbial species. Notably, S-equol exhibited positive correlations with several microbial taxa, including *Paenibacillus oryzisoli*, *Thiopseudomonas*, and *Chloroflexi* bacterium. Conversely, negative correlations were observed between S-equol and taxa such as *Citrobacter* sp. Lgbk14 and *Klebsiella* sp. HSTU Sny5. Additionally, inflammatory markers and oxidative stress indicators showed distinct associations with specific bacterial species, indicating associations between microbial composition and host redox and inflammatory status. These correlations suggest potential microbial contributors to both metabolic phenotypes and S-equol bioavailability in the context of OP.

Based on these findings, we applied a Random Forest algorithm to identify microbial features most strongly associated with OP status (Figure 3C). The top-ranked species included *Phocaeicola salanitronis*, *Cytobacillus gottheilii*, *Prevotella* sp. CAG255, and *Pediococcus acidilactici*, which contributed notably to the model’s classification accuracy. Of these, several species showed clear enrichment in the OP group, while others were more abundant in controls, reflecting disease-specific microbial signatures. To evaluate the diagnostic utility of the top discriminatory taxa, receiver operating characteristic (ROC) analysis was performed (Figure 3D). *Cytobacillus gottheilii* achieved the highest area under the curve (AUC = 0.8575), followed by *Pediococcus acidilactici* (AUC = 0.8077) and *Prevotella* sp. CAG255 (AUC = 0.7817), showing their potential as non-invasive biomarkers for identifying OP.

### Differential fecal metabolites and functional pathway enrichment analysis

**Figure 4:**
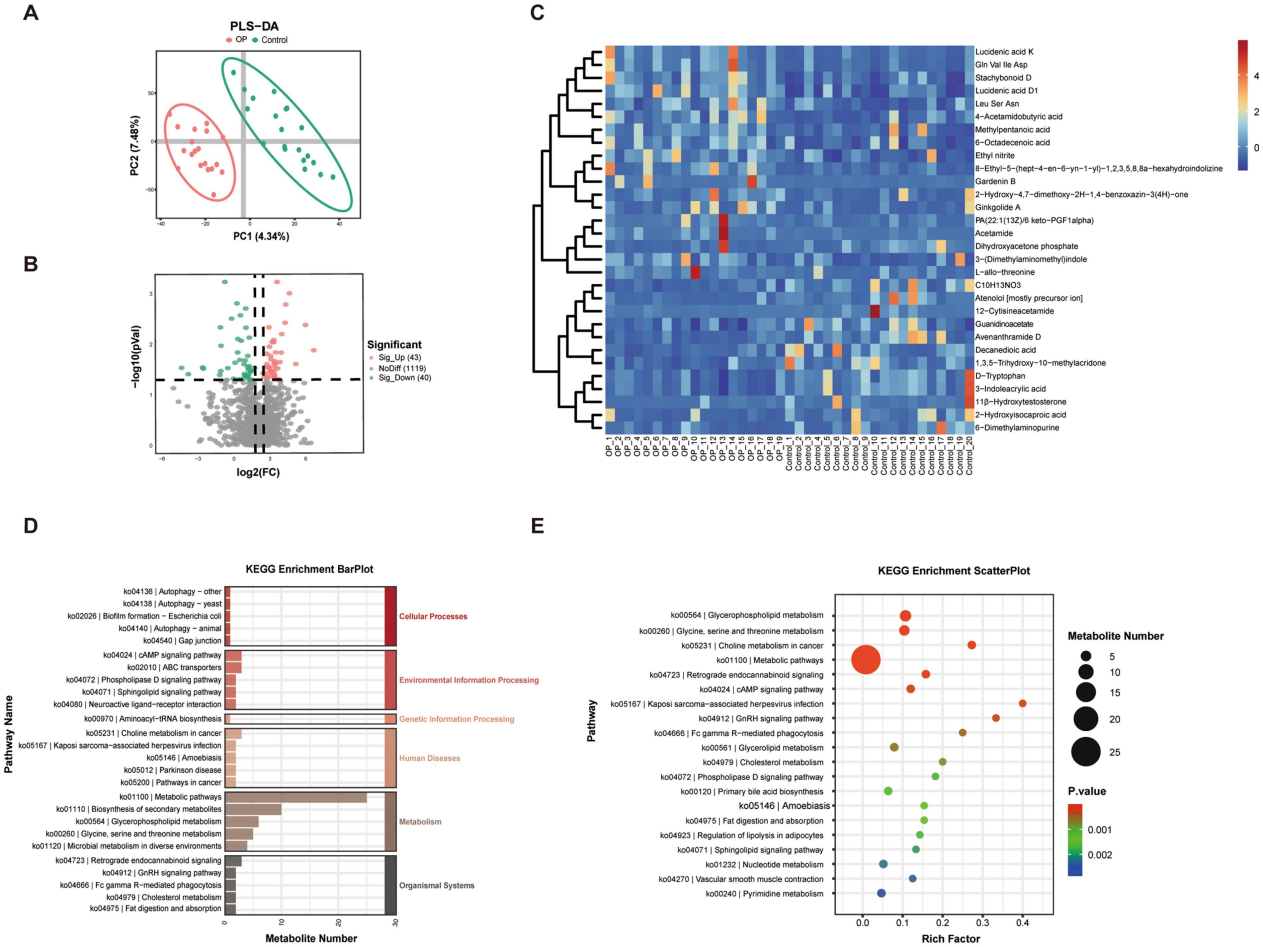
Multivariate and pathway enrichment analysis of differential metabolites between OP and control groups. **(A)** PLS-DA score plot showing clear separation between the OP and control groups based on serum metabolomic profiles. Each dot represents an individual sample; ellipses represent 95% confidence intervals. **(B)** Volcano plot illustrating significantly different metabolites between groups (OP vs. control). Metabolites with |log₂ fold change| > 1 and *p* < 0.05 were considered significant (red: upregulated in OP; green: downregulated). **(C)** Heatmap of differential metabolites, clustered by Pearson correlation. Rows represent metabolites and columns represent individual samples. The color gradient from blue to red indicates increasing metabolite intensity (log-transformed). **(D)** KEGG enrichment bar plot of differential metabolites. **(E)** KEGG enrichment bubble plot, where each bubble represents an enriched KEGG pathway. The size of the bubble reflects the number of metabolites enriched in that pathway, and the color indicates statistical significance.

To assess the global variation in fecal metabolite composition between osteoporotic individuals and controls, a partial least squares discriminant analysis (PLS-DA) was conducted. As shown in Figure 4A, the PLS-DA plot demonstrated a clear separation between the OP and control groups along the first two components (PC1 and PC2), which explained 4.34% and 7.48% of the total variance respectively, and it indicated that OP was accompanied by notable alterations in gut microbial metabolism, warranting further identification of differential fecal metabolites and associated biochemical pathways.

To further identify specific fecal metabolites that differed significantly between osteoporotic individuals and controls, differential analysis was conducted and visualized using a volcano plot. As shown in Figure 4B, a total of 83 metabolites showed significant differences between the OP and control groups. 43 upregulated metabolites (red dots) were predominantly enriched in the OP group, while 40 downregulated metabolites (green dots) were more abundant in control individuals. The majority of metabolites (gray dots, *n* = 1,119) did not exhibit statistically significant differences between the two groups. The identified differentially abundant metabolites provide a foundation for subsequent pathway enrichment and correlation analyses to explore their potential involvement in bone metabolism or S-equol production. To further visualize the expression patterns of the most significantly different metabolites, a hierarchical clustering heatmap was constructed based on the top-ranked differential features (Figure 4C). Each column represents an individual sample, and each row corresponds to a significantly dysregulated metabolite. The heatmap revealed distinct clustering patterns between the OP and control groups, supporting the separation observed in the PLS-DA and volcano plots. Several metabolites, including D-tryptophan, 3-indoleacrylic acid, decanedioic acid, and guanidinoacetate, exhibited markedly higher intensities in the control individuals. In contrast, several amino acid metabolites such as Gln-Val-Ile-Asp, Leu-Ser-Asn, acetamide, and avenanthramide D, were enriched in the OP individuals. The clear metabolic differentiation and specific fecal metabolites may be served as potential biomarkers or mechanistic indicators of gut metabolic dysbiosis in OP.

To explore potential functional implications, differential fecal metabolites between osteoporotic and control groups were mapped to the KEGG database. The KEGG bar plot categorizes the enriched pathways into six major functional classes, as showed in Figure 4D. Among these, metabolism-related pathways were the most dominant, particularly those involved in lipid and amino acid metabolism, underscoring their relevance to membrane lipid remodeling and nitrogen metabolism, which could contribute to osteoporotic pathophysiology. Complementarily, the KEGG scatter plot (Figure 4E) offers an overview of enrichment significance and the number of differential metabolites involved. Pathways with the highest enrichment scores and lowest *p*-values included glycerophospholipid metabolism, glycine/serine/threonine metabolism, and retrograde endocannabinoid signaling, showing a functional link to host-microbiota interactions and skeletal health. Additional pathways such as bile acid biosynthesis, gonadotropin-releasing hormone (GnRH) signaling, cAMP signaling, and sphingolipid signaling were also enriched, implicating alterations in hormonal regulation and immune signaling cascades.

### Key metabolite signatures between individuals with and without OP

**Figure 5:**
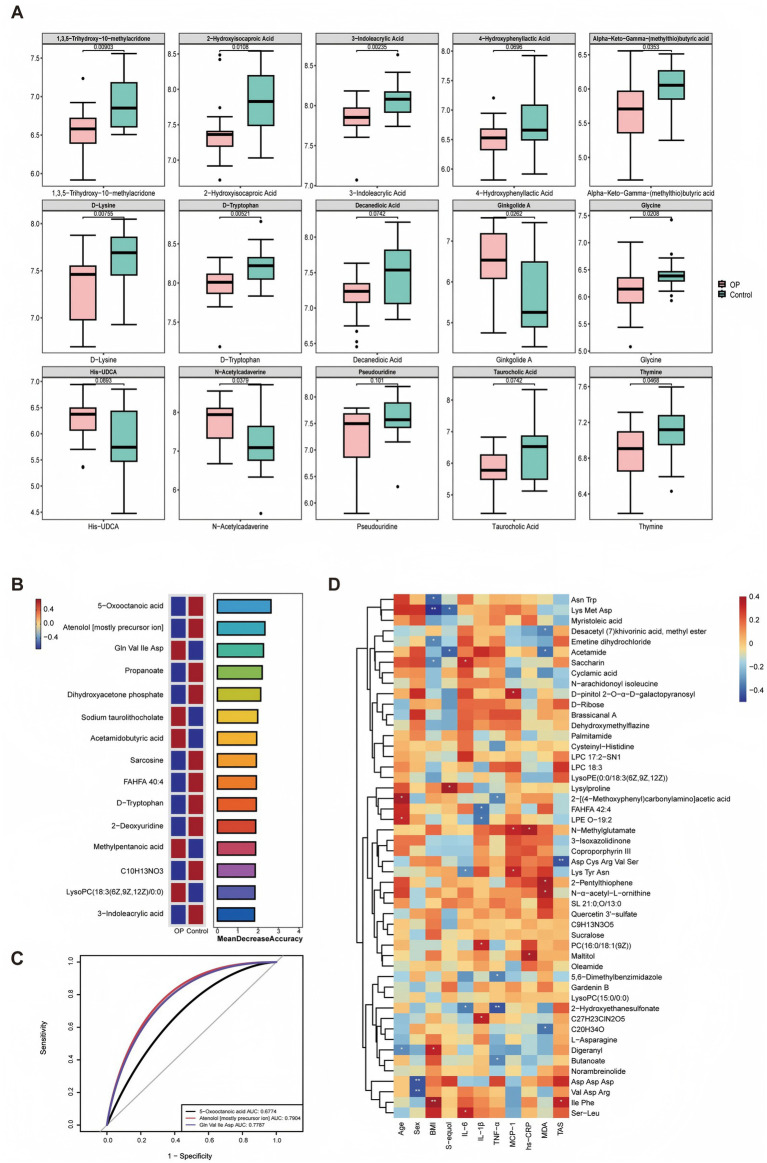
Identification of key serum metabolites associated with OP. **(A)** Boxplots showing significantly different serum metabolite levels between the OP and control groups (*n* = 19 vs. *n* = 20, respectively). Statistical significance was determined using Student’s *t*-test or Mann–Whitney *U* test, with *p* values adjusted as needed. **(B)** Top-ranked differential metabolites based on random forest analysis. The importance of each feature is reflected by the mean decrease accuracy value, and the relative abundance distribution between groups is indicated by the color blocks (blue: OP, red: control). **(C)** ROC curves showing diagnostic performance of representative metabolites in discriminating OP from control individuals. **(D)** Heatmap of Spearman correlation analysis between selected serum metabolites and clinical parameters. Blue to red color scale represents negative to positive correlation coefficients, respectively. *p < 0.05, **p < 0.01.

To further assess the group-specific differences in fecal metabolites, boxplot analysis was performed on 15 representative discriminatory metabolites between the OP and control groups (Figure 5A). Notably, several metabolites, such as D-tryptophan, 3-indoleacrylic acid, and 2-hydroxyindoleacetic acid, were present at lower levels in the OP group and were associated with differences in microbial tryptophan and amino acid metabolic profiles. To further identify key metabolites that best distinguish osteoporotic individuals from controls, a random forest classification model was applied. As shown in Figure 5A, the top 15 discriminatory metabolites were ranked according to their importance, measured by the mean decrease in classification accuracy. Among these, 5-oxooctanoic acid, atenolol, and Gln-Val-Ile-Asp, were identified as the most informative features contributing to the model’s predictive performance. Color-coding of relative abundance patterns indicated clear group-specific differences, with several metabolites markedly enriched in the OP group (red), while others showed higher levels in controls (blue). These metabolites are involved in diverse biological processes including lipid metabolism, bile acid conjugation, tryptophan degradation, and amino acid turnover, pathways that may be relevant to gut microbial activity and bone metabolism. To evaluate the diagnostic potential of key metabolites identified through random forest analysis, ROC curve analyses were conducted using the top three features ranked by MeanDecreaseGini. As shown in Figure 5C, atenolol exhibited the highest discriminative ability with an AUC of 0.7904, followed closely by Gln-Val-Ile-Asp (AUC = 0.7787) and 5-oxooctanoic acid (AUC = 0.6774).

To explore the potential clinical relevance of fecal metabolic alterations, we performed a correlation analysis between significantly different fecal metabolites and various host parameters, including serum S-equol, inflammatory cytokines, and oxidative stress markers (Figure 5D). Notably, S-equol showed negative correlations with Asn-Trp and acetamide, and a strong positive correlation with lysylproline, showing a potential involvement in modulating amino acid metabolism. Furthermore, inflammatory and oxidative stress markers exhibited significant correlations with some distinct metabolites, highlighting the complex interplay among host immune responses, oxidative stress, and microbial metabolic outputs.

### Correlation analysis between gut microbiota and metabolites

**Figure 6:**
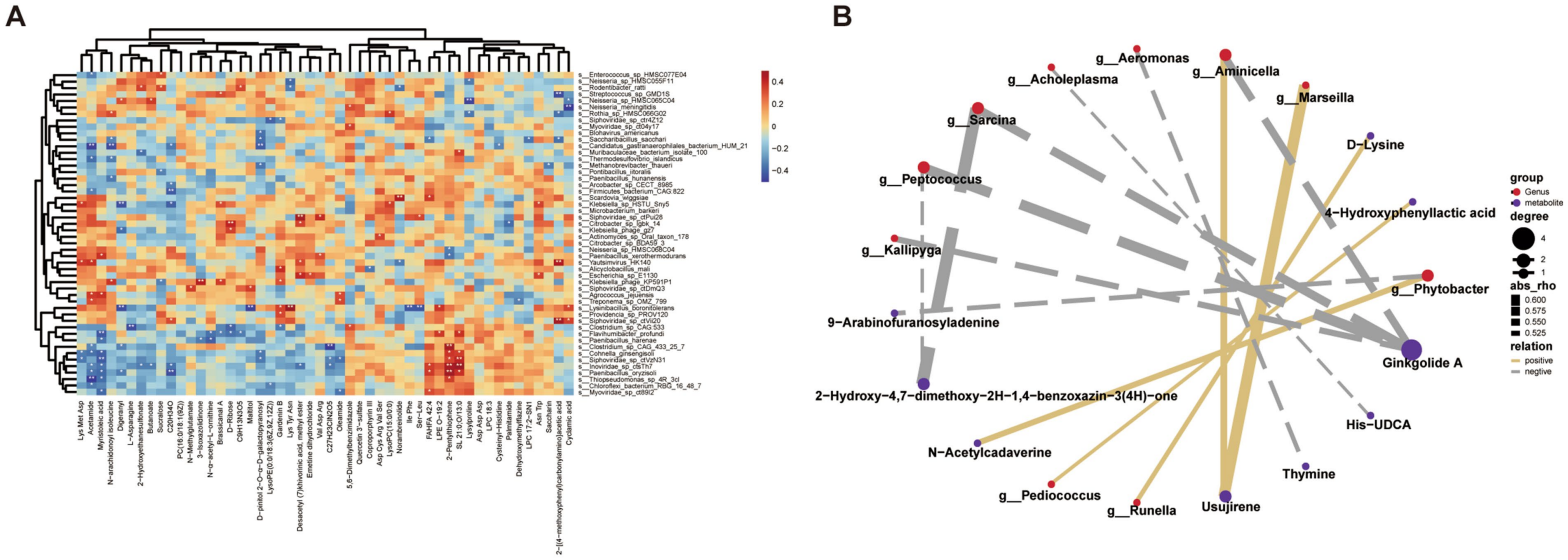
Correlation analysis between differential gut microbiota and serum metabolites in OP and control groups. **(A)** Heatmap illustrating the Spearman correlation coefficients between the top significantly different serum metabolites and gut microbial taxa (genus/species level) in the OP and control groups. The color scale denotes correlation strength (blue: negative, red: positive, and *p < 0.05, **p < 0.01). **(B)** Correlation network showing significantly correlated gut microbial genera (red nodes) and serum metabolites (purple nodes) (|rho| > 0.5, *p < 0.05). Node size corresponds to degree centrality, and edge width indicates correlation strength. Solid orange lines represent positive correlations, while dashed gray lines denote negative correlations.

To investigate the potential interplay between different gut microbial taxa and fecal metabolites, we performed Spearman correlation analyses and constructed a metabolite-microbe interaction network. As shown in Figures 6A,B multiple gut microbial species exhibited significant correlations with distinct metabolites including amino acid derivatives, plant secondary metabolites, and phospholipids. These findings highlight intricate connections between specific microbial taxa and host fecal metabolites, and may contribute to host metabolic phenotypes relevant to bone metabolism and warrant further mechanistic validation.

## Discussion

OP is a multifactorial metabolic bone disorder characterized by reduced bone mass, deterioration of trabecular microarchitecture, and increased skeletal fragility, which predisposes older adults to fractures (27). This study offers a multi-omics perspective on the relationship between gut microbiota, serum S-equol levels, fecal metabolites, and OP in residents aged ≥50 years from a community in Hainan Province, China’s only tropical island province. It demonstrated that individuals with OP had significantly lower serum S-equol levels, along with distinct alterations in gut microbial composition and fecal metabolites.

Firstly, we compared serum indicators between osteoporotic and control individuals, including S-equol, inflammatory cytokines, and oxidative stress-related markers. The most significant finding is that serum S-equol levels were markedly lower in individuals with OP compared to controls, supporting the hypothesis that reduced microbial equol production may be linked to bone loss. This aligns with earlier studies indicating the protective effects of S-equol on bone health through its estrogen receptor *β* agonism and antioxidant properties (12, 13). S-equol is a gut-derived metabolite produced from dietary soy isoflavones, particularly daidzein, through microbial transformation, and it exerts estrogen-like biological effects in humans (11). It is the only enantiomer of equol synthesized in the human body, and its production depends on specific intestinal bacteria such as *Slackia isoflavoniconvertens* and *Adlercreutzia equolifaciens*. These microbes express *β*-glucosidase enzymes that convert inactive glycosylated isoflavones into bioactive S-equol, which has a high binding affinity for estrogen receptor *β* and possesses antioxidant activity (15). Our findings indicate an association between lower serum S-equol levels and differences in gut microbiota composition and metabolomic profiles among individuals with osteoporosis.

In contrast, most biomarkers of inflammation and oxidative stress did not reach statistical significance, with the exception of a slight elevation of IL-1*β* in the OP group. However, we also observed that the levels of IL-1*β* and TNF-*α* in both groups were elevated above the ranges typically reported for healthy adults. This is anticipated in older adult population, who often exhibit chronic low-grade inflammation and subclinical comorbidities that contribute to higher circulating inflammatory markers (28). This suggests that low-grade inflammation, though present in aging population, may not be the primary differentiator between OP and non-OP individuals in our study population. Additionally, inflammatory markers and oxidative stress indicators were associated with specific bacterial species (e.g., *Akkermansia*), which have previously been reported to be involved in lipid metabolism, anti-inflammatory processes, and gut barrier maintenance (29, 30), suggesting potential relevance to host metabolic and mucosal homeostasis. These associations suggest that microbial variation may be linked to host redox and inflammatory status.

To investigate the microbial dysbiosis associated with OP, we performed integrated metagenomic analyses. While no significant differences were observed in *α*- or *β*-diversity indices between OP and control groups, several key bacterial taxa exhibited marked shifts, and most notably those involved in S-equol biosynthesis, such as *Faecalibacterium*, *Roseburia*, and *Blautia*, which showed lower relative abundances in the OP group. These genera are recognized producers of SCFAs and are known to modulate gut epithelial integrity and immune responses, which may indirectly influence isoflavone metabolism and S-equol production (31, 32). The correlation heatmap analysis (Figure 3B) further indicated that serum S-equol levels were positively correlated with several beneficial species depleted in OP, including *Paenibacillus oryzisoli*, *Thiopseudomonas*, and *Chloroflexi* bacterium. In parallel, S-equol concentrations exhibited significant correlations with several fecal metabolites acid-related to amino acids. Notably, S-equol was negatively associated with metabolites such as Asn-Trp and acetamide, but exhibited a strong positive correlation with lysylproline. Interestingly, the abundances of these metabolites displayed opposite patterns in OP individuals, in whom S-equol showed lower levels. Moreover, several key tryptophan-derived metabolites, including D-tryptophan and 3-indoleacrylic acid, were also observed at lower levels in the OP group compared with controls. Taken together, these findings suggest that lower S-equol levels in OP individuals were associated with differences in amino acid- and tryptophan-related metabolic profiles, thereby highlighting a possible association between gut-derived S-equol and bone health. Previous research has reported that 3-indoleacrylic acid, a microbiota-derived tryptophan metabolite, possesses anti-inflammatory and gut barrier-protective effects (33), which may act synergistically with S-equol to preserve bone homeostasis. These findings indicate associations among gut microbiota composition, serum S-equol levels, and fecal metabolic profiles in OP and underscore the relevance of microbial-metabolite interactions in the pathophysiology of age-related bone loss.

Beyond taxonomic composition, recent studies have emphasized functional interactions within microbial communities (34). In our co-occurrence network analysis, OP-enriched taxa formed tighter and more modular sub-networks, showing potential cooperative colonization or functional compensation under dysbiotic conditions. In contrast, taxa depleted in OP individuals exhibited weakened connections, indicating a potential loss of beneficial microbial interactions important for bone homeostasis (35–37). Moreover, opportunistic pathogens and inflammation-associated microbes, such as *Klebsiella pneumoniae* and members of *Gammaproteobacteria*, were significantly enriched in the OP group. These findings are consistent with previous reports associating microbial dysbiosis, increased gut permeability, and systemic inflammation with bone loss (38, 39). The concurrent reduction in SCFA-producing commensals likely contributes to impaired mucosal and metabolic homeostasis, further exacerbating bone deterioration.

In this study, untargeted fecal metabolomics revealed significant metabolic disturbances in individuals with OP, particularly within amino acid metabolism and tryptophan-derived pathways. Among the discriminatory metabolites, several tryptophan derivatives, including 3-indoleacrylic acid and 2-hydroxyindoleacetic acid, were markedly decreased in the OP group, indicating a potential impairment in microbial tryptophan catabolism. These metabolites are known for their anti-inflammatory and gut barrier–protective effects, which may play a role in maintaining bone homeostasis. Notably, correlation analyses demonstrated that S-equol was positively associated with several amino acid-related fecal metabolites, such as lysylproline and N-methylglutamate. These findings suggest that S-equol may serve as a regulatory nexus between isoflavone biotransformation and host amino acid metabolic networks. Furthermore, ROC analysis identified the Gln-Val-Ile-Asp peptide cluster as having the highest area under the curve (AUC = 0.7787), reflecting potential disturbances in amino acid metabolism relevant to OP pathophysiology. Collectively, these results reveal concurrent alterations in gut microbiota and metabolic features associated with lower serum S-equol levels.

Interestingly, the detection of atenolol, a non-endogenous antihypertensive agent, among the discriminatory metabolites suggests a possible comorbidity of hypertension in the osteoporotic group. This observation is in line with previous epidemiological evidence indicating an association between hypertension and increased OP risk, particularly in postmenopausal and older adult populations (40). Its presence in fecal samples likely reflects concurrent medication use and suggests a higher prevalence of hypertension among individuals with OP. Given the established links between hypertension, vascular dysfunction, and bone loss in older adults, future studies would benefit from incorporating more detailed clinical phenotyping and comprehensive medication histories. Additionally, 5-oxooctanoic acid, a lipid metabolism-related intermediate, exhibited moderate discriminatory power and may indicate gut microbiota-mediated disturbances in fatty acid oxidation and energy homeostasis. Given that fatty acid metabolic remodeling has been shown to influence bone mass during peri- and postmenopausal periods (41), this metabolite may represent an additional link between host energy metabolism and skeletal health.

To further elucidate the biological significance of the observed alterations in microbial composition and fecal metabolites in OP, KEGG pathway enrichment analysis was conducted on the differential metabolites. Several significantly enriched pathways were identified, including glycerophospholipid metabolism, glycine, serine and threonine metabolism, and choline metabolism. These pathways are fundamentally involved in lipid homeostasis, amino acid metabolism, and cellular signaling processes, all of which are critically linked to bone remodeling and skeletal integrity (14). Importantly, many of these metabolic pathways are known to be modulated by gut microbiota activity, including those responsible for the biosynthesis of S-equol, SCFAs, and other bone-active microbial metabolites (42). These findings align well with the results of our correlation analyses, which highlighted key associations between beneficial microbial taxa (e.g., *Faecalibacterium*, *Roseburia*) and amino acid-related or tryptophan-derived metabolites, showing a functional connection between microbial metabolism and host skeletal regulation. The convergence of microbiota profiling, metabolomic data, and KEGG enrichment supports the presence of a dysregulated microbiota-metabolite-bone axis in individuals with OP. In particular, disruptions in microbial lipid and amino acid metabolic pathways may underlie altered production of bone-protective metabolites such as S-equol and SCFAs, ultimately contributing to compromised bone homeostasis. This integrated evidence underscores the potential for targeting microbial metabolism as a novel strategy for OP prevention and therapy.

This study was conducted in a community-dwelling older adult population from Hainan Province, China’s only tropical island region, and employed a multi-dimensional approach to investigate the associations among S-equol, gut microbiota, fecal metabolites, and bone health. By integrating serum biomarkers with gut microbiota and metabolomic profiles, the present work provides a comprehensive view of the microbial-metabolic features associated with OP in older adults. Importantly, the distinct environmental, dietary, and lifestyle characteristics of Hainan offer a unique setting for exploring gut microbiota-bone interactions. These region-specific features may contribute to microbial and metabolic patterns that differ from those reported in temperate regions, thereby adding complementary evidence to existing studies predominantly conducted in non-tropical populations. Collectively, these findings highlight the value of incorporating geographical and environmental contexts into investigations of the gut-bone axis in aging populations.

However, several limitations should be acknowledged when interpreting the present findings. First, the cross-sectional design precludes any causal inference. Second, although the sample size was sufficient for an exploratory multi-omics analysis, it inevitably limited the statistical power to detect more subtle associations, particularly in subgroup analyses. Accordingly, the results should be interpreted as hypothesis-generating rather than definitive. Third, the association of this study were analyzed in univariate format due to our small sample size. We did not adjusted for potential confounders, such as detailed dietary intake, physical activity, medication intake, and others, that may have influences on our association estimations, future large cohort study should take these factors into considerations.

To further advance this field, prospective longitudinal studies with larger and more diverse cohorts are needed to validate the observed associations and to clarify their temporal and causal relationships. In parallel, functional investigations, including microbial gene expression profiling, targeted metabolite quantification, and mechanistic validation in animal models, will be essential to elucidate the biological pathways underlying the gut microbiota-metabolite-bone axis. From a translational perspective, interventions aimed at modulating S-equol producing microbes or their associated metabolic pathways may represent promising strategies for the prevention or management of OP in aging populations.

## Conclusion

In summary, this pilot study demonstrates that older adults with osteoporosis from a tropical community exhibit lower serum S-equol levels accompanied by distinct alterations in gut microbial composition and fecal metabolic profiles. These changes are characterized by lower abundance of specific SCFA-producing and isoflavone-transforming taxa, together with perturbations in amino acid- and tryptophan-related metabolic pathways. While the cross-sectional design precludes causal inference, the observed associations suggest that variations in microbial metabolic capacity may be linked to S-equol availability and bone health status in older adults. Our findings should be regarded as hypothesis-generating and provide a basis for future longitudinal and mechanistic studies to further clarify the role of gut microbiota-derived metabolites in osteoporosis, particularly in populations living under distinct dietary and environmental conditions.

## Data Availability

The data presented in this study are publicly available. The data can be found here: https://www.ebi.ac.uk/metabolights/MTBLS13855, accession number MTBLS13855.

## Supplementary material

SUPPLEMENTARY FIGURE S1 Gut microbial *α*-diversity assessed by Shannon and Simpson indices. Boxplots showing gut microbial *α*-diversity measured by the Shannon index **(A)** and Simpson index **(B)** in the OP and control groups. Boxes represent the interquartile range with the median indicated by the horizontal line, and whiskers represent the data range.

## References

[1] Liu Y Huang X Tang K Wu J Zhou J Bai H . Prevalence of osteoporosis and associated factors among Chinese adults: a systematic review and modelling study. J Glob Health. (2025) 15:04009. doi: 10.7189/jogh.15.04009, 39820179 PMC11737814

[2] Song S Guo Y Yang Y Fu D. Advances in pathogenesis and therapeutic strategies for osteoporosis. Pharmacol Ther. (2022) 237:108168. doi: 10.1016/j.pharmthera.2022.108168, 35283172

[3] Zeng Q Li N Wang Q Feng J Sun D Zhang Q . The prevalence of osteoporosis in China, a Nationwide, Multicenter Dxa survey. J Bone Miner Res. (2019) 34:1789–97. doi: 10.1002/jbmr.3757, 31067339

[4] Wang J Shu B Tang DZ Li CG Xie XW Jiang LJ . The prevalence of osteoporosis in China, a community based cohort study of osteoporosis. Front Public Health. (2023) 11:1084005. doi: 10.3389/fpubh.2023.1084005, 36875399 PMC9978786

[5] Si L Winzenberg TM Jiang Q Chen M Palmer AJ. Projection of osteoporosis-related fractures and costs in China: 2010-2050. Osteoporos Int. (2015) 26:1929–37. doi: 10.1007/s00198-015-3093-2, 25761729

[6] Barnsley J Buckland G Chan PE Ong A Ramos AS Baxter M . Pathophysiology and treatment of osteoporosis: challenges for clinical practice in older people. Aging Clin Exp Res. (2021) 33:759–73. doi: 10.1007/s40520-021-01817-y, 33742387 PMC8084810

[7] Pouresmaeili F Kamalidehghan B Kamarehei M Goh YM. A comprehensive overview on osteoporosis and its risk factors. Ther Clin Risk Manag. (2018) 14:2029–49. doi: 10.2147/TCRM.S138000, 30464484 PMC6225907

[8] Messina M Barnes S Setchell KD. Perspective: isoflavones-intriguing molecules but much remains to be learned about these soybean constituents. Adv Nutr. (2025) 16:100418. doi: 10.1016/j.advnut.2025.100418, 40157603 PMC12245442

[9] Cui Y Cai H Gao Y Dai Q Yang G Zheng W . Associations of dietary intakes of calcium, magnesium and soy isoflavones with osteoporotic fracture risk in postmenopausal women: a prospective study. J Nutr Sci. (2022) 11:e62. doi: 10.1017/jns.2022.52, 35992572 PMC9379929

[10] Miedziaszczyk M Maciejewski A Idasiak-Piechocka I Karczewski M Lacka K. Effects of isoflavonoid and vitamin D synergism on bone mineral density-a systematic and critical review. Nutrients. (2023) 15:5014. doi: 10.3390/nu15245014, 38140273 PMC10745652

[11] Seo H Seo H Lee SH Park Y. Receptor mediated biological activities of phytoestrogens. Int J Biol Macromol. (2024) 278:134320. doi: 10.1016/j.ijbiomac.2024.134320, 39084415

[12] Nishide Y Tadaishi M Kobori M Tousen Y Kato M Inada M . Possible role of S-Equol on bone loss via amelioration of inflammatory indices in ovariectomized mice. J Clin Biochem Nutr. (2013) 53:41–8. doi: 10.3164/jcbn.12-123, 23874069 PMC3705151

[13] Hu YC Huang TC Huang LW Cheng HL Hsieh BS Chang KL. S-Equol ameliorates menopausal osteoarthritis in rats through reducing oxidative stress and cartilage degradation. Nutrients. (2024) 16:2364. doi: 10.3390/nu16142364, 39064807 PMC11280421

[14] Xu Z Xu J Li S Cui H Zhang G Ni X . S-Equol enhances osteoblastic bone formation and prevents bone loss through Opg/Rankl via the Pi3k/Akt pathway in streptozotocin-induced diabetic rats. Front Nutr. (2022) 9:986192. doi: 10.3389/fnut.2022.986192, 36337646 PMC9633996

[15] Yuan JP Wang JH Liu X. Metabolism of dietary soy isoflavones to equol by human intestinal microflora--implications for health. Mol Nutr Food Res. (2007) 51:765–81. doi: 10.1002/mnfr.200600262, 17579894

[16] Mayo B Vazquez L Florez AB. Equol: a bacterial metabolite from the daidzein isoflavone and its presumed beneficial health effects. Nutrients. (2019) 11:2231. doi: 10.3390/nu11092231, 31527435 PMC6770660

[17] Xiao H Wang Y Chen Y Chen R Yang C Geng B . Gut-bone axis research: unveiling the impact of gut microbiota on postmenopausal osteoporosis and osteoclasts through mendelian randomization. Front Endocrinol (Lausanne). (2024) 15:1419566. doi: 10.3389/fendo.2024.1419566, 38883609 PMC11176613

[18] Zhang YW Song PR Wang SC Liu H Shi ZM Su JC. Diets intervene osteoporosis via gut-bone Axis. Gut Microbes. (2024) 16:2295432. doi: 10.1080/19490976.2023.2295432, 38174650 PMC10773645

[19] Zhang YW Wu Y Liu XF Chen X Su JC. Targeting the gut microbiota-related metabolites for osteoporosis: the inextricable connection of gut-bone axis. Ageing Res Rev. (2024) 94:102196. doi: 10.1016/j.arr.2024.102196, 38218463

[20] He Y Chen Y. The potential mechanism of the microbiota-gut-bone Axis in osteoporosis: a review. Osteoporos Int. (2022) 33:2495–506. doi: 10.1007/s00198-022-06557-x, 36169678

[21] Seely KD Kotelko CA Douglas H Bealer B Brooks AE. The human gut microbiota: a key mediator of osteoporosis and osteogenesis. Int J Mol Sci. (2021) 22:9452. doi: 10.3390/ijms22179452, 34502371 PMC8431678

[22] Qin Q Yan S Yang Y Chen J Yan H Li T . The relationship between osteoporosis and intestinal microbes in the Henan Province of China. Front Cell Dev Biol. (2021) 9:752990. doi: 10.3389/fcell.2021.752990, 34869341 PMC8638085

[23] Wang H Liu J Wu Z Zhao Y Cao M Shi B . Gut microbiota signatures and Fecal metabolites in postmenopausal women with osteoporosis. Gut Pathog. (2023) 15:33. doi: 10.1186/s13099-023-00553-0, 37415173 PMC10324172

[24] Wang Z Wang W Wang Y Hu H Wang B Zhu W . Mapping gut microbiota and metabolite alterations in patients with postmenopausal osteoporosis in the Beijing Community of China. Eur J Med Res. (2025) 30:539. doi: 10.1186/s40001-025-02795-x, 40597307 PMC12211186

[25] Thalapalliyil K Bobby Z Dorairajan G Toi PC. Maternal systemic Iron homeostasis in diabetes-in-pregnancy: a cross-sectional analytical study from a tertiary health Care Centre of South India. Saudi Med J. (2025) 46:1017–23. doi: 10.15537/smj.2025.46.9.20250320, 40897421 PMC12441910

[26] Amarnath SS Kumar V Das SL. Classification of osteoporosis. Indian J Orthop. (2023) 57:49–54. doi: 10.1007/s43465-023-01058-3, 38107823 PMC10721754

[27] Ye C Ebeling P Kline G. Osteoporosis. Lancet. (2025) 406:2003–2016. doi: 10.1016/S0140-6736(25)01385-6, 40946719

[28] Franceschi C Garagnani P Parini P Giuliani C Santoro A. Inflammaging: a new immune-metabolic viewpoint for age-related diseases. Nat Rev Endocrinol. (2018) 14:576–90. doi: 10.1038/s41574-018-0059-4, 30046148

[29] Derrien M Vaughan EE Plugge CM de Vos WM. *Akkermansia muciniphila* gen. Nov., sp. nov., a human intestinal mucin-degrading bacterium. Int J Syst Evol Microbiol. (2004) 54:1469–76. doi: 10.1099/ijs.0.02873-0, 15388697

[30] Li C Stražar M Mohamed AMT Pacheco JA Walker RL Lebar T . Gut microbiome and metabolome profiling in Framingham heart study reveals cholesterol-metabolizing Bacteria. Cell. (2024) 187:1834–52.e19. doi: 10.1016/j.cell.2024.03.014, 38569543 PMC11071153

[31] Feng B Lu J Han Y Han Y Qiu X Zeng Z. The role of short-chain fatty acids in the regulation of osteoporosis: new perspectives from gut microbiota to bone health: a review. Medicine (Baltimore). (2024) 103:e39471. doi: 10.1097/MD.0000000000039471, 39183408 PMC11346881

[32] Ohlsson C Sjogren K. Effects of the gut microbiota on bone mass. Trends Endocrinol Metab. (2015) 26:69–74. doi: 10.1016/j.tem.2014.11.004, 25497348

[33] Zelante T Iannitti RG Cunha C De Luca A Giovannini G Pieraccini G . Tryptophan catabolites from microbiota engage aryl hydrocarbon receptor and balance mucosal reactivity via interleukin-22. Immunity. (2013) 39:372–85. doi: 10.1016/j.immuni.2013.08.003, 23973224

[34] Kuo YJ Chen CJ Hussain B Tsai HC Hsu GJ Chen JS . Inferring bacterial community interactions and functionalities associated with osteopenia and osteoporosis in Taiwanese postmenopausal women. Microorganisms. (2023) 11:234. doi: 10.3390/microorganisms11020234, 36838199 PMC9959971

[35] Chu X Xing H Chao M Xie P Jiang L. Gut microbiota modulation in osteoporosis: probiotics, prebiotics, and natural compounds. Meta. (2025) 15:301. doi: 10.3390/metabo15050301, 40422878 PMC12113025

[36] Mehta M Hodgson E Reimer RA Gabel L. Gut microbiome-targeted therapies and bone health across the lifespan: a scoping review. Crit Rev Food Sci Nutr. (2025) 65:4759–72. doi: 10.1080/10408398.2024.2397459, 39216013

[37] Ticinesi A Siniscalchi C Meschi T Nouvenne A. Gut microbiome and bone health: update on mechanisms, clinical correlations, and possible treatment strategies. Osteoporos Int. (2025) 36:167–91. doi: 10.1007/s00198-024-07320-0, 39643654

[38] Indrio F Salatto A. Gut Microbiota-Bone Axis. Ann Nutr Metab. (2025) 81:47–56. doi: 10.1159/000541999, 39848230

[39] Pacifici R. Bone remodeling and the microbiome. Cold Spring Harb Perspect Med. (2018) 8:a031203. doi: 10.1101/cshperspect.a031203 28847904 PMC5880157

[40] Chai H Ge J Li L Li J Ye Y. Hypertension is associated with osteoporosis: a case-control study in Chinese postmenopausal women. BMC Musculoskelet Disord. (2021) 22:253. doi: 10.1186/s12891-021-04124-9, 33678168 PMC7938480

[41] Gong R Xiao HM Zhang YH Zhao Q Su KJ Lin X . Identification and functional characterization of metabolites for bone mass in peri- and postmenopausal Chinese women. J Clin Endocrinol Metab. (2021) 106:e3159–77. doi: 10.1210/clinem/dgab146, 33693744 PMC8277206

[42] Yumol JL Gittings W de Souza RJ Ward WE. A systematic review and Meta-analysis of the effects of probiotics on bone outcomes in rodent models. J Bone Miner Res. (2024) 40:100–13. doi: 10.1093/jbmr/zjae187, 39545776 PMC11700591

