# Supplementary figures and images for: Exploratory multi-omics analysis of gut microbiota and fecal metabolites in relation to serum S-equol levels in older adults with osteoporosis from a tropical community: a pilot study
Source: Front Nutr. 2026 Feb 18;13:1784894. doi: 10.3389/fnut.2026.1784894 (PMC12957214; doi:10.3389/fnut.2026.1784894)

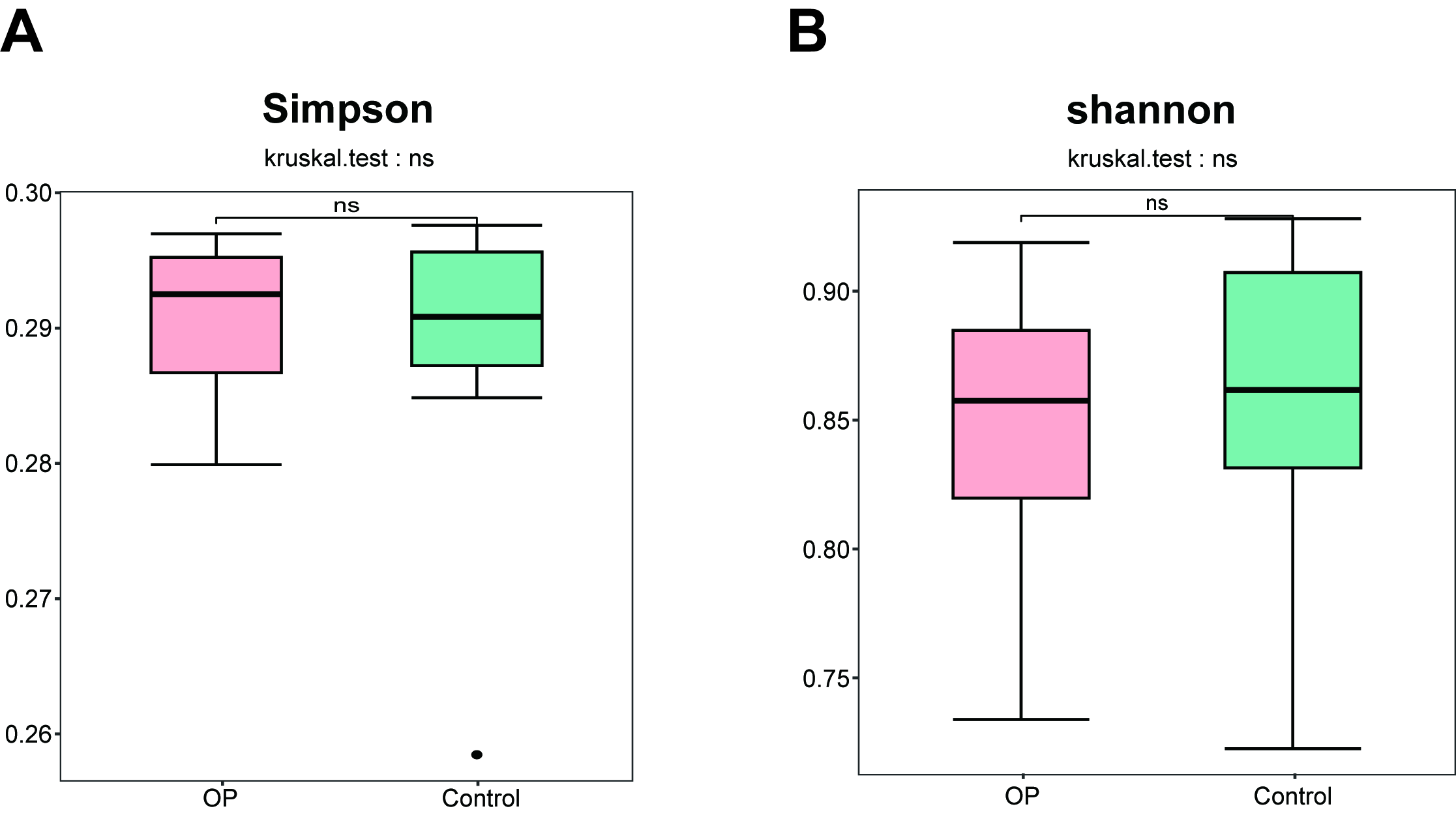

Supplement: SUPPLEMENTARY FIGURE S1 — Gut microbial α-diversity assessed by Shannon and Simpson indices. Boxplots showing gut microbial α-diversity measured by the Shannon index (A) and Simpson index (B) in the OP and control groups. Boxes represent the interquartile range with the median indicated by the horizontal line, and whiskers represent the data range. [file Image_1.TIF]
